# Remote Collaborative Depression Care Program for Adolescents in Araucanía Region, Chile: Randomized Controlled Trial

**DOI:** 10.2196/jmir.8021

**Published:** 2018-01-31

**Authors:** Vania Martínez, Graciela Rojas, Pablo Martínez, Pedro Zitko, Matías Irarrázaval, Carolina Luttges, Ricardo Araya

**Affiliations:** ^1^ Centro de Medicina Reproductiva y Desarrollo Integral del Adolescente Facultad de Medicina Universidad de Chile Santiago Chile; ^2^ Instituto Milenio para la Investigación en Depresión y Personalidad Santiago Chile; ^3^ Departamento de Psiquiatría y Salud Mental Hospital Clínico Universidad de Chile Santiago Chile; ^4^ Centro de Innovación en Tecnologías de la Información para Aplicaciones Sociales Universidad de Santiago de Chile Santiago Chile; ^5^ Escuela de Psicología Facultad de Humanidades Universidad de Santiago de Chile Santiago Chile; ^6^ Health Service & Population Research Department Institute of Psychiatry, Psychology & Neuroscience King's College London London United Kingdom; ^7^ Unidad de Estudios Asistenciales Complejo Asistencial Barros Luco Santiago Chile; ^8^ Mental Health and Substance Use Unit Pan American Health Organization/World Health Organization Washington, DC United States; ^9^ Centre for Global Mental Health and Primary Care Research, Health Service & Population Research Department Institute of Psychiatry, Psychology & Neuroscience King’s College London London United Kingdom

**Keywords:** primary health care, depression, adolescents, Internet, telemedicine, medically underserved area

## Abstract

**Background:**

Despite evidence on efficacious interventions, a great proportion of depressed adolescents do not receive evidence-based treatment and have no access to specialized mental health care. Remote collaborative depression care (RCDC) may help to reduce the gap between needs and specialized mental health services.

**Objective:**

The objective of this study was to assess the feasibility, acceptability, and effectiveness of an RCDC intervention for adolescents with major depressive disorder (MDD) living in the Araucanía Region, Chile.

**Methods:**

A cluster randomized, assessor-blind trial was carried out at 16 primary care centers in the Araucanía Region, Chile. Before randomization, all participating primary care teams were trained in clinical guidelines for the treatment of adolescent depression. Adolescents (N=143; 13-19 years) with MDD were recruited. The intervention group (RCDC, N=65) received a 3-month RCDC treatment that included continuous remote supervision by psychiatrists located in Santiago, Chile’s capital city, through shared electronic health records (SEHR) and phone patient monitoring. The control group (enhanced usual care or EUC; N=78) received EUC by clinicians who were encouraged to follow clinical guidelines. Recruitment and response rates and the use of the SEHR system were registered; patient adherence and satisfaction with the treatment and clinician satisfaction with RCDC were assessed at 12-week follow-up; and depressive symptoms and health-related quality of life (HRQoL) were evaluated at baseline and 12-weeks follow-up.

**Results:**

More than 60.3% (143/237) of the original estimated sample size was recruited, and a response rate of 90.9% (130/143) was achieved at 12-week follow-up. A mean (SD) of 3.5 (4.0) messages per patient were written on the SEHR system by primary care teams. A third of the patients showed an optimal adherence to psychopharmacological treatment, and adolescents in the RCDC intervention group were more satisfied with psychological assistance than those in EUC group. Primary care clinicians were satisfied with the RCDC intervention, valuing its usefulness. There were no significant differences in depressive symptoms or HRQoL between groups. Satisfaction with psychological care, in both groups, was related to a significant change in depressive symptomatology at 12-weeks follow-up (beta=−4.3, 95% CI −7.2 to −1.3).

**Conclusions:**

This is the first trial of its kind in Latin America that includes adolescents from vulnerable backgrounds, with an intervention that proved to be feasible and well accepted by both patients and primary care clinicians. Design and implementation issues may explain similar effectiveness across arms. The effectiveness of the intervention seems to be comparable with an already nationwide established treatment program that proved to be highly efficacious under controlled conditions.

**Trial Registration:**

ClinicalTrials.gov: NCT01860443; https://clinicaltrials.gov/ct2/show/NCT01860443 (Archived by WebCite at http://www.webcitation.org/6wafMKlTY)

## Introduction

### Adolescent Depression in Primary Care

At least 8% of adolescents attending primary care centers have probable major depressive disorder (MDD) [1]. Characterized by marked symptoms of low mood and anhedonia [2], adolescent MDD is associated with suicidal ideation [3,4], subsequent alcohol and illicit drug use [5], and poor living standards and worse mental health in adulthood [6,7]. Despite the burden of adolescent depression and the availability of efficacious interventions [8-10], a great proportion of adolescents with MDD do not receive evidence-based treatment or get no treatment at all [11,12].

Primary care has a major role in the delivery of depression treatment for adolescents [13]. The collaborative care model, defined as the team-driven, population-focused, measurement-guided, and evidence-based provision of care [14], has been regarded as one of the best approaches in integrated behavioral health [15]. Its effectiveness in treating adolescents with MDD has also been demonstrated, achieving significant improvements in depressive symptoms and global functioning [16,17]. However, most evidence comes from trials conducted in the United States [16-20], with multifaceted interventions to provide mental health care to mostly non-Hispanic white and urban adolescents [17-20].

### The Chilean Case: the Use of Technologies and Collaborative Care for Depression

Chile, a developing country with a growing income inequality [21], has made substantial efforts during the past decades to improve mental health services, providing one of the first examples of an evidence-based depression intervention being scaled up in resource-constrained settings [22,23]. Yet, primary care providers’ barriers to treating depression [24] coupled with the uneven geographical distribution of mental health resources, which especially affects low-income people living in hard-to-reach areas [25], make the Chilean case worth studying.

Being a country with an above-average digital connectivity [26], the use of information and communication technologies (ICTs) in Chile may help overcome the inequity of access to mental health resources. According to international evidence, ICTs have made it possible to effectively assist underserved populations through remote collaborative care programs [27,28], obtaining results comparable with face-to-face interventions in the management of depression that have mostly benefited white rural adults [29,30]. No adolescent-specific electronic mental health collaborative care for depression has been tested before.

This study tested the feasibility, acceptability, and effectiveness of a remote collaborative care program delivered by primary and specialized mental health teams to enhance the management of adolescent MDD in the Araucanía Region, an underserved area of Chile.

## Methods

### Study Design

This was an assessor-blind, 2-group, cluster randomized (1:1 ratio) clinical trial, carried out in the Araucanía Region, Chile. Randomization was conducted using computer-generated random numbers.

### Setting, Participants, and Eligibility Criteria

Araucanía Region is the poorest administrative division of Chile (20.7% of households living below the poverty line) and has the highest proportion of ethnic minorities and rural population [31,32]. Compared with the national average, children and adolescents of the region live in precarious material conditions and suffer from the highest suicide rates, with 6.7 deaths due to suicide per 100,000 inhabitants [32]. Temuco, the capital city of Araucanía Region, is located 380 miles south of Santiago, Chile’s capital city.

This clinical trial was conducted in 16 primary care centers located in the Araucanía Region. These centers were required to have an Internet connection, receive no psychiatric consulting services for children or adolescents, and ensure the recruitment of 15 depressed adolescents over a 13-month period. The health centers entered the study after being authorized by their boards of directors and receiving the informed consent of at least one family physician and one psychologist from each health team.

Adolescents aged between 13 and 19 years with suspected depression were invited (face-to-face) to participate in the study by their primary care teams; the informed consent of those over 18 years and the informed assent of minors along with their parents’ or primary caregivers’ informed consent were obtained. A child and adolescent psychiatrist in Santiago verified over the telephone that the adolescents could be diagnosed with depression and not psychotic depression, bipolar disorder, comorbidity with substances and or or alcohol dependence, or suicide risk requiring immediate specialized treatment. To do this, the semistructured clinical interview MINI-KID (Mini-International Neuropsychiatric Interview For Children and Adolescents) was used, which enables researchers to make a diagnostic assessment in children and adolescents according to the criteria of International Statistical Classification of Diseases and Related Health Problems, 10th Revision, and Diagnostic and Statistical Manual of Mental Disorders, 4th Edition [33].

### Interventions

#### Computer-Based Remote Depression Training for Health Teams

Before the random assignment, all the health teams received a Web-based training to improve their early detection of depression in adolescents and increase their access to timely and effective treatment. The training program was designed by 2 psychiatrists, 1 psychologist, and 1 social worker from Universidad de Chile’s Faculty of Medicine. It was aimed at presenting a comprehensive approach to depression and suicide in adolescence and a critical assessment of the key recommendations issued by the Chilean Ministry of Health to overcome problems usually encountered in clinical practice when treating depression in adolescents.

This program was delivered over 30 hours, during which teaching activities were conducted using audiovisual material to present clinical cases. In addition, the participating health teams were encouraged to participate actively in discussion forums. The participants’ learning outcomes were assessed with online written worksheets in which they were requested to apply their clinical judgment to tackle the clinical cases presented earlier and a multiple-choice test that considered the material covered in the program.

#### Remote Collaborative Depression Care for Adolescents

A remote collaborative depression care (RCDC) program for adolescents introduced shared electronic health records (SEHR) to improve communication between the clinicians in the Araucanía Region and the team of child and adolescent mental health specialists of Universidad de Chile’s Faculty of Medicine, operating via an Internet platform in a secure virtual environment provided by the faculty. The SEHR functioned as a discussion forum allowing for personalized, confidential, and real-time interaction between the primary care teams and the specialists to assist the former during the diagnostic process and the treatment of the acute phase of the disorder.

In addition, after each patient’s data was entered into his or her SEHR, a structured phone monitoring system hosted by Universidad de Chile’s Faculty of Medicine was implemented by a psychologist trained in Santiago. Phone calls were done at 1, 2, 3, 6, and 9 weeks post baseline assessment, each lasting for about 5 to 10 min per adolescent and his/her primary caregiver. Contents of this component included monitoring of symptoms and adherence to pharmacological treatment, assessment of side effects of medications, evaluation of the quality of the therapeutic relationship, and agreements on the goals and tasks of the therapy. If patients could not be contacted, several attempts were made on different days of the week and times of the day, with no restrictions imposed on the number of attempts. This made it possible to triangulate the information obtained from the primary care teams, thus providing the specialists with other elements to be included in the online assistance sessions.

#### Enhanced Usual Care

The clinicians in the Araucanía Region centers that were randomly assigned to the control group were encouraged to follow the recommendations of the Ministry of Health of Chile for the management of adolescent depression and also received the main conclusions of the baseline diagnostic assessment.

### Assessments

The feasibility of the project was assessed in terms of achieving the recruitment targets, the attrition rates at 12 weeks, and the use of SEHR by the primary care teams.

The acceptability of the intervention was assessed by comparing rates of pharmacological adherence and user satisfaction with the components of the treatment displayed by each group at 12-week follow-up. In addition, data reflecting the satisfaction of the primary care teams with the RCDC were analyzed.

Outcomes regarding the acceptability of the intervention were assessed as follows: Pharmacological adherence was assessed with a brief structured interview, asking patients whether their physician prescribed medications and whether those medications were taken correctly during the last 4 days and, if not, with the reasons for nonadherence. User satisfaction with treatment was evaluated through a self-reported questionnaire with a 7-point Likert scale, which included 5 items to rate treatment, facilities, medical care, psychological care, and nonprofessional staff treatment. For clinicians in the active group, the use of SEHR was noted and an RCDC satisfaction questionnaire was administered.

The primary outcome with respect to the effectiveness of the RCDC was assessed with mean Beck Depression Inventory (BDI) [34] scores at 12 weeks after treatment started. The BDI is a self-reported questionnaire for assessing depressive symptoms in people aged 13 years and older [34], which has been previously used in Chile [35]. The secondary outcome was change in health-related quality of life (HRQoL) at 12 weeks measured through the KIDSCREEN-27 questionnaire [36]. The KIDSCREEN-27 questionnaire measures the frequency or intensity of behaviors, feelings, or attitudes of the adolescent about different aspects of his/her everyday life during the previous week [36]. This questionnaire has been validated in Chile [37].

Patient baseline data and outcomes at 12-week follow-up were evaluated via telephone by a trained consultant at Universidad de Chile’s Faculty of Medicine, who was blinded to treatment allocation.

### Statistical Analysis

#### Sample Size

On the basis of previous studies [35,38], a sample size of 237 depressed adolescents was needed to detect a difference between groups in terms of an expected reduction in mean Beck scores of 3.2 points across arms at 12 weeks, representing approximately one-third of SD difference. This sample size has a power of 82%, with a one-sided alpha of .05.

#### Data Analysis

Descriptive analyses for relevant variables were conducted for the total sample and for each group separately to assess the balance across arms after randomization. Fisher exact test and independent samples *t* test were used to compare differences in patient adherence and treatment satisfaction between groups. Primary and secondary outcomes are described in terms of mean and SD pre- and postintervention. The magnitude of the effect of the intervention was evaluated using regression models, which included a random effect to account for intracluster correlations within primary care centers. Univariate and multivariate models were employed. Multivariate models included the baseline outcome value and variables that were not well balanced after randomization. Furthermore, using the same regression models, we explored the relevance of other health care features on the magnitude of change on the primary outcome. Regression coefficients are presented with their respective 95% CIs. Because missing primary outcome values were less than 7%, a sensitivity analysis was not conducted. All analyses were conducted using R 3.1.1 (R Foundation for Statistical Computing, Vienna, Austria) and its package lme4 for multilevel analysis. This trial has been reported in accordance to the CONSORT-EHEALTH guidelines [39].

### Ethics

The study was approved by the Committee of Human Research Ethics of Universidad de Chile’s Faculty of Medicine (Nº 080-2012), the Committee of Scientific Ethics of the North Araucanía Health Service, and the Committee of Scientific Ethics of the South Araucanía Health Service. The study allowed for participants’ voluntary withdrawal with no adverse consequences. The local teams treating the participants, both in the active and in the control groups, were informed of cases in which high suicide risk or any other high-risk situations were detected for them to carry out the necessary actions.

## Results

### Sample Characteristics

The sample comprised 143 adolescents with MDD. Most of the participants were women (81.1%), aged 15.4 years (SD 1.6), living with both parents (47.6%), and of middle-low socioeconomic status (45.5%). Of the participants, 46.9% reported having had at least one mental health problem in their life, whereas 37.8% reported the same regarding one of their close relatives. Table 1 shows the baseline sociodemographic characteristics and clinical information of the sample, stratified by treatment group (enhanced usual care or EUC or RCDC). Significant differences exist between the groups in terms of socioeconomic status (*P*=.03).

### Feasibility

#### Recruitment and Follow-Up

**Figure 1:**
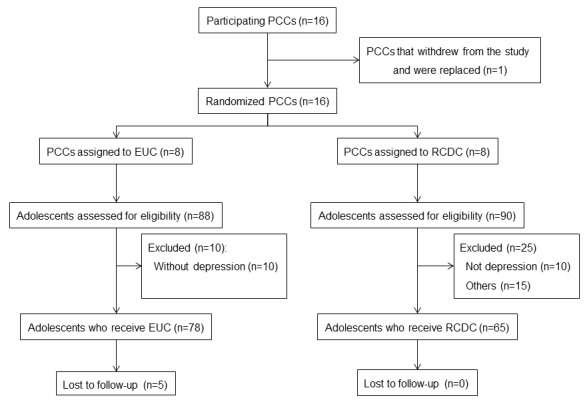
Flow diagram. EUC: enhanced usual care; PCCs: primary care centers; RCDC: remote collaborative depression care.

In total, 16 primary care centers participated in the study; one of these withdrew and was replaced. The participating health care centers were able to identify 178 adolescents with suspected depression and managed to recruit 143 eligible individuals, which represents 60.3% of the initially projected sample size. Of the participants, 45.5% received the RCDC intervention, whereas 54.5% received EUC (Figure 1).

At 12-week follow-up, 5 cases (6.4%) in EUC were lost to follow-up, and there was no lost to follow-up in RCDC (Figure 1).

#### Use of Shared Electronic Health Records

The team of child and adolescent mental health specialists wrote on the Internet platform a mean (SD) of 9.9 (4.2) times per patient, ranging from 3 to 28 messages, throughout the study period (12 weeks). The experts helped by providing continuous training for the health staff, general recommendations about actions and strategies to be implemented, and specific case management guidelines. They covered the following areas: assessment of depressive symptoms, medical treatment of depression (pharmacological and nonpharmacological), and psychosocial interventions. Their specific indications covered aspects such as confidentiality and its limits, sexual and reproductive health counseling, and working with family and school support networks.

Regarding the primary care teams, it was observed that those in the RCDC group wrote a mean (SD) of 3.5 (4.0) messages per patient. In 13 of the cases assigned to the RCDC group, 20% of the adolescents receiving RCDC, the clinicians from primary care teams wrote no messages, whereas in 16.7% of cases they did so only once.

### Acceptability

#### Patient Adherence and Treatment Satisfaction

In general, nearly one-third of the sample displayed a regular intake of the psychoactive medications prescribed (n=44). No significant differences were observed across groups at 12-week follow-up in terms of adherence to pharmacological treatment (Fisher exact test *P* value=.98). Satisfaction outcomes reached 6 points on a scale from 1 to 7, with no differences across arms. Satisfaction with psychological care was the only aspect of treatment satisfaction that displayed a significant difference between the groups (Wilcoxon rank-sum test *P* value=.04), with the RCDC intervention scoring higher (Table 2).

In addition, patients in the active group received a mean (SD) of 3.8 (1.0) monitoring phone calls lasting 5 to 10 min each. On a scale from 1 (worst) to 7 (best), patients assigned a mean (SD) score of 6.2 (1.2) to the usefulness of these calls and 6.2 (1.2) to how comfortable they felt with the phone monitoring.

**Table 1:** Baseline characteristics of the sample.

Variables		EUC^a^ (N=78)	RCDC^b^ (N=65)	*P* value for between-groups differences
Sex (female), mean (SD)		64 (82.1)	52 (80.0)	.83^c^
Age in years, mean (SD)		15.6 (1.7)	15.2 (1.5)	.20^d^
Mapuche ethnicity^e^, n (%)		11 (14)	13 (20)	.38^c^
Living in a rural area, n (%)		11 (14)	14 (22)	.27^c^
**Living with, n (%)**				.88^c^
	Both parents	34 (44)	34 (52)	
	Mother only	22 (28)	16 (25)	
	Mother and her partner	8 (10)	5 (8)	
	Father only	4 (5)	5 (8)	
	Father and his partner	1 (1)	0 (0)	
	Other relatives	8 (10)	5 (8)	
	Other nonrelatives	1 (1)	0 (0)	
**Socioeconomic status, n (%)**				.03^c^
	Low	11 (14)	16 (25)	
	Mid-low	33 (42)	32 (49)	
	Middle	22 (28)	16 (25)	
	Mid-high	8 (10)	1 (2)	
	High	4 (5)	0 (0)	
Years of schooling, mean (SD)		9.3 (2)	8.9 (2)	.10^d^
Personal history of mental illness, n (%)		33 (42)	34 (52)	.24^c^
Family history of mental illness, n (%)		34 (44)	20 (31)	.12^c^
Beck Depression Inventory score, mean (SD)		27.1 (9)	27.8 (10)	.65^f^
Perceived health^g^, mean (SD)		3.6 (1)	3.6 (1)	.97^d^
Physical well-being^h^, mean (SD)		30.9 (9)	31.4 (9)	.78^f^
Psychological well-being^h^, mean (SD)		29.6 (10)	30.2 (10)	.98^d^
Autonomy and parents^h^, mean (SD)		37.6 (8)	39.1 (9)	.67^f^
Peers and social support^h^, mean (SD)		40.1 (13)	40.3 (14)	.92^f^
School environment^h^, mean (SD)		37.8 (8)	38.2 (8)	.82^f^
Health-related quality of life index^i^, mean (SD)		33.8 (7)	33.5 (7)	.84^f^

^a^EUC: enhanced usual care.

^b^RCDC: remote collaborative depression care.

^c^Fisher exact test *P* value.

^d^Wilcoxon rank-sum test *P* value.

^e^Mapuche are the indigenous people of the region.

^f^Student *t* test for unequal variances.

^g^KIDSCREEN-27 item score.

^h^KIDSCREEN-27 dimension score.

^i^Uses 10 items derived from the 27-item version of KIDSCREEN.

**Table 2:** Patient satisfaction with treatment at 12-week follow-up in the remote collaborative depression care (RCDC) and the usual care group.

Outcomes	EUC^a^		RCDC		Wilcoxon rank-sum test *P* value
	N^b^	Mean^c^ (SD)	N^b^	Mean^c^ (SD)	
Satisfaction with treatment	70	6.0 (1.1)	63	6.2 (1.2)	.19
Satisfaction with facilities	70	6.2 (1.0)	63	6.3 (1.0)	.45
Satisfaction with medical care	70	6.4 (1.2)	63	6.7 (0.8)	.08
Satisfaction with psychological care	69	6.4 (0.9)	61	6.7 (0.7)	.04
Satisfaction with nonprofessional staff treatment	69	6.2 (1.1)	63	6.4 (0.9)	.65

^a^EUC: enhanced usual care.

^b^Cases providing complete data and analyzed.

^c^Possible scores ranging from 1 (worst) to 7 (best).

#### Clinician Satisfaction With Remote Collaborative Depression Care

The primary care teams in the active group assessed the following aspects of the RCDC intervention: (1) usefulness for clinical work—mean (SD) score 6.3 (1.0), ranging from 3 to 7 on a scale from 1 (worst) to 7 (best), mode 7; (2) usefulness for patients—mean score 6.5 (0.7), range 4 to 7, mode 7; and (3) comfort level of the Internet platform—mean score 6.0 (1), range 4 to 7, mode 7.

In addition, the main positive aspects, difficulties, and suggestions regarding the RCDC voiced by the primary care clinicians in the active group are summarized in Textbox 1.

### Effectiveness

#### Depressive Symptoms and Health-Related Quality of Life

No significant differences were observed across arms at 12-week follow-up in terms of depressive symptomatology or HRQoL before and after adjusting for baseline values and when introducing socioeconomic variables (Table 3).

**Main positive aspects, difficulties, and suggestions regarding the remote collaborative depression care (RCDC) intervention stated by clinicians in the active group.**

Positive aspects

- The preintervention training was useful.
- The Internet platform is an innovative resource.
- It is useful to receive timely specialized support.
- The phone assessment and monitoring of the patients contributes to their positive perception of the treatment and fosters their trust in the local health team.

Difficulties

- Clinicians’ times for accessing the Internet platform were not scheduled
- Lack of interest in mental health displayed by some physicians
- High turnover of professionals in local health care centers
- Depressed adolescents tend to resist interventions
- Insufficient communication with patients and their parents in phone monitoring
- Very complex cases with limited follow-up time
- Medical work receives more support than psychotherapeutic work

Suggestions

- Ensuring the continuity of interventions of this type
- Better coordination between local and specialized teams
- Facilitating the internal coordination of the local team
- More user-friendly Internet platform
- Conducting face-to-face or videoconference assistance sessions
- Assigning and scheduling local equipment use for local teams to fulfill tasks required by RCDC
- Fostering the participation of the whole health care team in distance assistance sessions
- Incorporating email alerts to be sent to clinicians

**Table 3:** Magnitude of the effect of the intervention on depressive symptoms and health-related quality of life.

Outcomes	Analyzed^a^, n (%)	Nonadjusted difference between means (95% CI)^b^	Adjusted difference between means^c^ (95% CI)
Beck Depression Inventory score	138 (0.97)	1.1 (−2.9 to 5.2)	1.5 (−2.4 to 5.6)
Perceived health^d^	134 (0.94)	−0.1 (−0.4 to 0.2)	−0.2 (−0.4 to 0.1)
Physical well-being^e^	134 (0.94)	1.7 (−2.1 to 5.4)	2.3 (−0.8 to 5.7)
Psychological well-being^e^	134 (0.94)	0.8 (−3.9 to 5.8)	1.0 (−3.4 to 5.8)
Autonomy and parents^e^	134 (0.94)	−0.7 (−4.2 to 2.5)	−0.1 (−3.1 to 2.9)
Peers and social support^e^	134 (0.94)	−1.3 (−5.6 to 3.5)	−0.2 (−4.3 to 3.8)
School environment^e^	109 (0.76)	−3.5 (−7.4 to 0.7)	−3.1 (−7.1 to 1.0)
Health-related quality of life index^f^	110 (0.77)	1.0 (−2.8 to 4.8)	1.2 (−3.2 to 5.2)

^a^Number of patients who provided outcome data at 12-week follow-up and percentage of full outcome data.

^b^Effect magnitude estimated with Markov Chain Monte Carlo procedures.

^c^Adjusted according to the baseline measurement and socioeconomic status differences observed.

^d^KIDSCREEN-27 item score.

^e^KIDSCREEN-27 dimension score.

^f^Uses 10 items derived from the 27-item version of KIDSCREEN.

**Table 4:** Adjusted regression model for change in depressive symptomatology at 12-week follow-up (N=130).

Variables	Depressive symptomatology^a^ at 12 weeks (95% CI)
Constant	32.0 (2.8-61.1)
Treatment group^b^	−8.0 (−40.0 to 24.0)
Initial depressive symptomatology^c^	−0.5 (−0.7 to −0.3)
Age	−0.1 (−1.4 to 1.2)
Sex	−0.1 (−5.3 to 5.1)
Ethnicity	−1.0 (−6.6 to 4.6)
Rurality	1.1 (−4.4 to 6.6)
Satisfaction with the psychological care received	−4.3 (−7.2 to −1.3)
Satisfaction with the psychological care received by treatment group	1.7 (−3.1 to 6.5)

^a^Beck Depression Inventory score at 12-week follow-up.

^b^Enhanced usual care or remote collaborative depression care.

^c^Baseline Beck Depression Inventory score.

#### Model for Change in Depressive Symptoms at 12-Week Follow-Up

Changes in depressive symptomatology at 12-week follow-up were explored with a regression model that considered the group to which the participants belonged, their baseline depressive symptomatology (according to BDI score), sex, age, ethnicity, rurality, and satisfaction with the psychological care received. Results show that (1) for each extra point in baseline depressive symptomatology (according to BDI scores), a reduction of 0.5 points in depressive symptomatology is expected at 12 weeks, keeping the value of the other variables constant and (2) for each additional point in satisfaction with the psychological care received, a reduction of 4.3 points in depressive symptomatology is expected at 12 weeks, keeping the value of the other variables constant. The interaction between the groups to which participants were randomized and their satisfaction with the care received was not significant (Table 4).

## Discussion

### Principal Findings

This clinical trial, conducted in 16 primary care centers located in vulnerable areas, far from the Chile’s capital city, achieved recruitment rates of 60% of the initially projected sample and high response rates at 12-week follow-up (90.9%). The RCDC intervention significantly surpassed EUC in terms of user satisfaction with psychological treatment. Phone monitoring, a component of the intervention, was well received by the adolescents, and the primary care teams perceived that the intervention was useful and provided them with innovative tools for the comprehensive management of adolescent depression.

However, over one-third of the clinicians of the primary care teams had little or no participation on the Internet platform, and only one-third of the patients displayed an adequate adherence to the pharmacological treatment. Clinicians who participated in the RCDC group identified some obstacles in primary mental health care that may compromise the integrity of the intervention. Finally, at 12-week follow-up, RCDC for the treatment of adolescent depression did appear to have equivalent effectiveness compared with EUC, achieving comparable levels of depressive symptoms and HRQoL. Overall, more severe cases seemed to benefit more from treatment, as well as those who felt more satisfied with the psychological care received.

### Strengths of the Study

This is the first report of a cluster randomized trial in a Latin American country that tests the feasibility, acceptability, and effectiveness of an ICT-assisted collaborative depression care intervention for the management of depressed adolescents from vulnerable backgrounds in remote areas lacking specialized mental health services.

Being able to train primary care teams and remotely implementing a complex intervention involving multiple components (RCDC) in several health care centers in a highly vulnerable area constitutes a major achievement, especially considering that the intervention was well received by the participants, increasing local teams’ trust and fostering a positive perception of the adolescent depression treatment provided.

Therefore, this study contributes to scientific knowledge in multiple ways: not only does it respond to the marked lack of research equality in the field of depression in vulnerable populations [40], but it also addresses the sore need to generate mental health–related learning opportunities aimed at primary care professionals in Latin America and the Caribbean [41] and helps tackle the limited access to current scientific and technological advances available to health care teams and individuals from remote areas, thus expanding clinicians’ problem-solving toolkit.

### Limitations of the Study

However, this study has several design and implementation limitations that need be solved before conducting a larger clinical trial with suitable statistical power to draw conclusive results for the assessment of the effectiveness of the intervention.

#### Baseline Characteristics of the Primary Care Clinics Not Assessed

As the groups were not matched according to the characteristics of the participating primary care teams, there is a possibility that EUC practices performed better regarding depression management than RCDC practices before the project started. Additionally, the initial training of EUC clinicians might have improved their ability to treat depression. Both the characteristics of the participating health care providers and the robustness of the usual care provided have been identified to play a role in the effects of similar interventions [15,42].

#### Inadequate Statistical Power

Only 60% of the initially projected sample was recruited. The most likely explanation of this is the high turnover of clinicians in remote areas and the service pressure in usual practice, which limited the health care teams’ ability to identify mental health problems in the population that they serve. To solve this issue, the researchers tried several strategies, such as expanding the recruitment period from 13 to 22 months, carefully reviewing the processes for detecting depressed adolescents in health care centers to strengthen their comprehensive assessment (eg, in sexual and reproductive health checkups conducted by midwives), establishing contact with nearby schools, and training personnel to detect and refer potentially depressed students.

#### Integrating Innovations Into Real-World Primary Care Settings

The use of the SEHR was a highly innovative tool aimed at improving the communication between health teams; however, it was not integrated into the daily routine of the participating physicians, and a limited time scheduled for this activity had to be negotiated with the local authorities. Moreover, the primary care teams were working in a context of high patient demand and regular staff turnover. Thus, registering patient information in the SEHR may have been experienced as burdensome by some of the physicians, partly explaining the low use of this system.

Over the course of the study, the researchers tried to complement the SEHR with more frequent in-person assistance to strengthen the bond and increase coordination between the local teams and specialists. However, this trial aimed to be pragmatic and to simulate real-life conditions rather than implement a highly complex, multifaceted intervention with high involvement of qualified study personnel, as those conducted in the United States [16,17]. In future implementations, if the integration of the SEHR into the currently working information system is not possible, it will be necessary, at least, to define protected time for using the Internet platform, explore elements that could make it more user-friendly, and possibly incorporate videoconferencing functionalities, depending on resource availability.

#### Patients’ Preferences and Staff Stability

The low percentage of patients who displayed satisfactory adherence to the pharmacological treatment prescribed may compromise the integrity of the intervention, although this issue must be contextualized. Studies conducted in other depressed populations in Chile highlight the role of beliefs and attitudes toward medication in treatment adherence[43], and it is plausible that adolescents coming from highly vulnerable areas—as those participating in this study—are specially prone to noncompliance because of a combination of biological, psychological, and social factors.[44]. Moreover, the high turnover of clinicians in remote areas, especially physicians, along with the difficulties of training the incoming health care personnel could have resulted in a level of antidepressant prescription higher than that recommended by physicians without suitable training for managing adolescent depression.

It is noteworthy that one of the differences between the groups in terms of user satisfaction concerned psychological treatment and that this was a significant predictor for a decrease in depressive symptoms at 12-week follow-up in both groups. This component of both interventions was provided by the most stable personnel in Chilean primary care teams—psychologists. Therefore, psychologists could play a more relevant role in future interventions of this type. However, it is worthwhile to mention that as no other treatment process outcome was assessed in this trial, it is not clear what may have been the cause(s) of these differences favoring the RCDC intervention in terms of satisfaction and why this apparent advantage did not translate into differences in depressive symptoms across groups. As such, future studies of this type must integrate such assessments.

#### Other Issues

This study used a short follow-up period at the end of acute phase of treatment according to national guidelines [45], whereas studies employing longer intervention and follow-up periods have reported that collaborative care for depressed adolescents is effective [16,17,19]. Finally, it is known that depression management in primary care settings is suboptimal [11], and RCDC was further hampered by barriers related to patient (low consultation and adherence rates), provider (lack of time/interest to participate), and system issues (high clinician turnover). Therefore, the RCDC intervention may have lacked intensity and or or duration to cause the desired effect. Finally, it seems the RCDC group had a higher proportion of adolescents from more vulnerable backgrounds, with significant differences in socioeconomic status but also observable differences in terms of ethnicity and rurality. The intervention has not been adapted to treat this vulnerable population, and although evidence shows that school-aged children from minority ethnic groups may be at higher risk for depression [46], collaborative depression care programs have been shown to reduce racial disparities in adults [47].

### Implications for Practice and Research

The implementation of this clinical trial in primary care centers in a vulnerable region of Chile was hindered by some structural characteristics of health services in the country. The high turnover level observed in health care teams, particularly involving physicians, prompts the need to develop a continuous distance training system that enables health care professionals who have recently joined a center to manage depressed adolescents. Likewise, the greater stability of psychologists in remote health care teams could make it possible to provide them with more intensive training in effective psychotherapy approaches for treating adolescent depression. Also, given their continuity, future initiatives could explore their role as case managers in charge of conducting a more thorough patient follow-up.

On the other hand, one of the main complaints received regarding the intervention concerned the user-friendliness of the Internet platform, the lack of time available to input case data and coordinate the teams, and the need to have specialists working in person with the primary care teams. In this respect, many primary care centers have electronic patient records; therefore, if specialists could establish contact using this medium, the intervention would become more user-friendly while also saving the local teams some time.

Research on collaborative care for the management of depression in adolescents is relatively scarce and limited to the United States. This clinical trial, carried out in a Latin American country, shows that these programs can be implemented in resource-poor settings and with vulnerable populations, reaching adequate acceptability levels among health care teams and patients. However, this study also highlights the difficulties involved in carrying out research in remote areas where the service pressure is high. The low levels of identification of depression conspired against achieving the targeted sample size.

### Conclusions

It is feasible to implement a remote collaborative program for the management of depression among vulnerable adolescents from areas with poor access to specialized mental health services. The intervention was shown to be acceptable for the participants and showed a promising way to reduce the mental health care gap in remote areas. Before conducting a future clinical trial with enough statistical power to assess the program’s effects on users’ depressive symptoms and HRQoL, it is necessary to improve recruitment strategies, promote more interaction between local teams and specialists through the Internet platform, and test innovative strategies to prevent staff turnover in remote areas from affecting the abilities and joint work of health care teams. Nonetheless, the effectiveness of the intervention seems to be comparable with an already nationwide established treatment program that proved to be highly efficacious under controlled conditions [22,23].

## Appendix

Multimedia Appendix 1 CONSORT-EHEALTH checklist (V 1.6.1).

## Abbreviations

BDI: Beck Depression Inventory
EUC: enhanced usual care
HRQoL: health-related quality of life
ICTs: information and communication technologies
MDD: major depressive disorder
RCDC: remote collaborative depression care
SEHR: shared electronic health record

## References

[1] Wright DR, Katon WJ, Ludman E, McCauley E, Oliver M, Lindenbaum J, Richardson LP (2016). Association of adolescent depressive symptoms with health care utilization and payer-incurred expenditures. Acad Pediatr.

[2] American Psychiatric Association (2013). Diagnostic and Statistical Manual of Mental Disorders, Fifth Edition.

[3] Strandheim A, Bjerkeset O, Gunnell D, Bjørnelv S, Holmen TL, Bentzen N (2014). Risk factors for suicidal thoughts in adolescence--a prospective cohort study: the Young-HUNT study. Br Med J Open.

[4] McManama O'Brien KH, Becker SJ, Spirito A, Simon V, Prinstein MJ (2014). Differentiating adolescent suicide attempters from ideators: examining the interaction between depression severity and alcohol use. Suicide Life Threat Behav.

[5] Conway KP, Swendsen J, Husky MM, He J, Merikangas KR (2016). Association of lifetime mental disorders and subsequent alcohol and illicit drug use: results from the National Comorbidity Survey-Adolescent Supplement. J Am Acad Child Adolesc Psychiatry.

[6] McLeod GF, Horwood LJ, Fergusson DM (2016). Adolescent depression, adult mental health and psychosocial outcomes at 30 and 35 years. Psychol Med.

[7] Gibb SJ, Fergusson DM, Horwood LJ (2010). Burden of psychiatric disorder in young adulthood and life outcomes at age 30. Br J Psychiatry.

[8] Zhou X, Hetrick SE, Cuijpers P, Qin B, Barth J, Whittington CJ, Cohen D, Del Giovane C, Liu Y, Michael KD, Zhang Y, Weisz JR, Xie P (2015). Comparative efficacy and acceptability of psychotherapies for depression in children and adolescents: a systematic review and network meta-analysis. World Psychiatry.

[9] Qin B, Zhang Y, Zhou X, Cheng P, Liu Y, Chen J, Fu Y, Luo Q, Xie P (2014). Selective serotonin reuptake inhibitors versus tricyclic antidepressants in young patients: a meta-analysis of efficacy and acceptability. Clin Ther.

[10] Cipriani A, Zhou X, Del Giovane C, Hetrick SE, Qin B, Whittington C, Coghill D, Zhang Y, Hazell P, Leucht S, Cuijpers P, Pu J, Cohen D, Ravindran AV, Liu Y, Michael KD, Yang L, Liu L, Xie P (2016). Comparative efficacy and tolerability of antidepressants for major depressive disorder in children and adolescents: a network meta-analysis. Lancet.

[11] Mojtabai R, Olfson M, Han B (2016). National trends in the prevalence and treatment of depression in adolescents and young adults. Pediatrics.

[12] Soria-Saucedo R, Walter HJ, Cabral H, England MJ, Kazis LE (2016). Receipt of evidence-based pharmacotherapy and psychotherapy among children and adolescents with new diagnoses of depression. Psychiatr Serv.

[13] Campo JV, Bridge JA, Fontanella CA (2015). Access to mental health services: implementing an integrated solution. J Am Med Assoc Pediatr.

[14] (2016). Psychiatry.

[15] Asarnow JR, Rozenman M, Wiblin J, Zeltzer L (2015). Integrated Medical-Behavioral Care Compared With Usual Primary Care for Child and Adolescent Behavioral Health: A Meta-analysis. JAMA Pediatr.

[16] Asarnow JR, Jaycox LH, Duan N, LaBorde AP, Rea MM, Murray P, Anderson M, Landon C, Tang L, Wells KB (2005). Effectiveness of a quality improvement intervention for adolescent depression in primary care clinics: a randomized controlled trial. J Am Med Assoc.

[17] Richardson LP, Ludman E, McCauley E, Lindenbaum J, Larison C, Zhou C, Clarke G, Brent D, Katon W (2014). Collaborative care for adolescents with depression in primary care: a randomized clinical trial. J Am Med Assoc.

[18] Clarke G, Debar L, Lynch F, Powell J, Gale J, O'Connor E, Ludman E, Bush T, Lin EH, Von Korff M, Hertert S (2005). A randomized effectiveness trial of brief cognitive-behavioral therapy for depressed adolescents receiving antidepressant medication. J Am Acad Child Adolesc Psychiatry.

[19] Richardson L, McCauley E, Katon W (2009). Collaborative care for adolescent depression: a pilot study. Gen Hosp Psychiatry.

[20] Wright DR, Haaland WL, Ludman E, McCauley E, Lindenbaum J, Richardson LP (2016). The costs and cost-effectiveness of collaborative care for adolescents with depression in primary care settings. J Am Med Assoc Pediatr.

[21] Organisation for Economic Development and Cooperation (2015). In It together: Why Less Inequality Benefits All.

[22] Araya R, Rojas G, Fritsch R, Gaete J, Rojas M, Simon G, Peters TJ (2003). Treating depression in primary care in low-income women in Santiago, Chile: a randomised controlled trial. Lancet.

[23] Araya R, Flynn T, Rojas G, Fritsch R, Simon G (2006). Cost-effectiveness of a primary care treatment program for depression in low-income women in Santiago, Chile. Am J Psychiatry.

[24] Vöhringer PA, Jimenez MI, Igor MA, Fores GA, Correa MO, Sullivan MC, Holtzman NS, Whitham EA, Barroilhet SA, Alvear K, Logvinenko T, Kent DM, Ghaemi NS (2013). Detecting mood disorder in resource-limited primary care settings: comparison of a self-administered screening tool to general practitioner assessment. J Med Screen.

[25] World Health Organization, Ministry of Health of Chile (2014). WHO-AIMS Report on Mental Health System in Chile.

[26] Huawei Technologies Co, Ltd (2017). Huawei.

[27] Fortney JC, Pyne JM, Turner EE, Farris KM, Normoyle TM, Avery MD, Hilty DM, Unützer J (2015). Telepsychiatry integration of mental health services into rural primary care settings. Int Rev Psychiatry.

[28] Hilty DM, Ferrer DC, Parish MB, Johnston B, Callahan EJ, Yellowlees PM (2013). The effectiveness of telemental health: a 2013 review. Telemed J E Health.

[29] Fortney JC, Pyne JM, Edlund MJ, Williams DK, Robinson DE, Mittal D, Henderson KL (2007). A randomized trial of telemedicine-based collaborative care for depression. J Gen Intern Med.

[30] Fortney JC, Pyne JM, Mouden SB, Mittal D, Hudson TJ, Schroeder GW, Williams DK, Bynum CA, Mattox R, Rost KM (2013). Practice-based versus telemedicine-based collaborative care for depression in rural federally qualified health centers: a pragmatic randomized comparative effectiveness trial. Am J Psychiatry.

[31] (2016). Ministerio de Desarrollo Social.

[32] Días S, Santibañez D, Cortés A, Raczynski G, Contreras N, Bozo N (2016). Observatorio Niñez y Adolescencia.

[33] Sheehan D, Sheehan K, Shytle R, Janavs Juris, Bannon Yvonne, Rogers Jamison E, Milo Karen M, Stock Saundra L, Wilkinson Berney (2010). Reliability and validity of the Mini International Neuropsychiatric Interview for Children and Adolescents (MINI-KID). J Clin Psychiatry.

[34] Beck AT, Steer RA, Carbin MG (1988). Psychometric properties of the Beck Depression Inventory: twenty-five years of evaluation. Clin Psychol Rev.

[35] Alvarado R, Vega J, Sanhueza G, Muñoz MG (2005). Evaluación del programa para la detección, diagnóstico y tratamiento integral de la depresión en atención primaria, en Chile. Rev Panam Salud Publica.

[36] KIDSCREEN Group Europe (2006). The KIDSCREEN Questionnaires. Quality of Life Questionnaires for Children and Adolescents-Handbook.

[37] Sepúlveda PR, Molina GT, Molina CR, Martínez NV, González AE, Montaño ER, Hidalgo-Rasmussen C (2013). [Validation of an instrument to measure health-related quality of life in Chilean children and adolescents]. Rev Med Chil.

[38] Fritsch R, Araya R, Solís J, Montt E, Pilowsky D, Rojas G (2007). Ensayo clínico aleatorizado de farmacoterapia con monitorización telefónica para mejorar el tratamiento de la depresión en la atención primaria en Santiago, Chile. Rev Méd Chile.

[39] Eysenbach G, CONSORT-EHEALTH Group (2011). CONSORT-EHEALTH: improving and standardizing evaluation reports of Web-based and mobile health interventions. J Med Internet Res.

[40] Perrino T, Beardslee W, Bernal G, Brincks A, Cruden G, Howe G, Murry V, Pantin H, Prado G, Sandler I, Brown CH (2015). Toward scientific equity for the prevention of depression and depressive symptoms in vulnerable youth. Prev Sci.

[41] (2013). Pan American Health Organization.

[42] Shirazi M, Lonka K, Parikh SV, Ristner G, Alaeddini F, Sadeghi M, Wahlstrom R (2013). A tailored educational intervention improves doctor's performance in managing depression: a randomized controlled trial. J Eval Clin Pract.

[43] Rojas G, Santelices M, Martínez P, Tomicic A, Reinel M, Olhaberry M, Krause M (2015). Barreras de acceso a tratamiento de la depresión posparto en Centros de Atención Primaria de la Región Metropolitana: un estudio cualitativo. Rev Méd Chile.

[44] Pacheco P B, Aránguiz G C (2011). Factores relacionados a la adherencia a tratamiento en adolescentes con depresión. Rev Chil Neuro-psiquiatr.

[45] Ministerio de Salud de Chile (2013). Normassalud.

[46] Stirling K, Toumbourou JW, Rowland B (2015). Community factors influencing child and adolescent depression: A systematic review and meta-analysis. Aust N Z J Psychiatry.

[47] Davis TD, Deen T, Bryant-Bedell K, Tate V, Fortney J (2011). Does minority racial-ethnic status moderate outcomes of collaborative care for depression?. Psychiatr Serv.

